# Comparison of clipping and endoscopic band ligation treatment for the colonic wall: an in vivo animal study

**DOI:** 10.1016/j.igie.2023.07.005

**Published:** 2023-07-15

**Authors:** Junnosuke Hayasaka, Daisuke Kikuchi, Masayuki Kemi, Shu Hoteya

**Affiliations:** 1 Department of Gastroenterology, Toranomon Hospital, Tokyo, Japan; 2 Fukushima Medical Device Industry Promotion Agency, Fukushima Medical Device Development Support Centre, Division of Safety and Biological Compatibility Assessment Veterinarian, Fukushima, Japan

## Abstract

**Background and aims:**

Although clipping is a simple and widely used treatment for colonic diverticular bleeding, endoscopic band ligation (EBL) has recently been reported to be more effective than clipping in preventing recurrent bleeding. However, the pathologic effects of these treatments on the colonic mucosa remain unclear. This study compares the effects of those treatments on the colonic mucosa using a pig model.

**Methods:**

Six clipping procedures and 6 EBL procedures each were performed on the normal colon of the pig, with the clipping and EBL sites separated by approximately 1 cm. The procedure was performed by an expert endoscopist. The pig was killed 10 days after the procedures, and the specimens were compared for pathologic evaluation of necrosis, fibrosis, and disruption of the muscularis mucosae of the colonic mucosa to determine the effects on the colonic mucosa.

**Results:**

All bands and clips remained in the procedure site; no perforation was observed at either site. Necrosis was significantly less in the clip group (*P* = .002). Disruption of the muscularis mucosae was less common in the clip group, but the difference was not significant (*P* = .181). Fibrosis of the submucosa was less in the clip group (0 of 6) than in the EBL group (6 of 6) (*P* = .002). Fibrosis extending to the serosa was less in the clip group but was not statistically significant: 0 (0%) in the clip group and 4 (66.7%) in the EBL group (*P* = .061).

**Conclusions:**

The impact on the colonic mucosa is small and localized with clipping but large and extensive with EBL.

Colonic diverticular bleeding (CDB) is the most common cause of acute lower GI bleeding.^1^, ^2^, ^3^ Although CDB often results spontaneously in hemostasis,^4^^,^^5^ a high early recurrent bleeding rate of 26.7% to 75% has been reported with conservative treatment.^6^, ^7^, ^8^

In definitive CDB, treatment with stigmata of recent hemorrhage and endoscopic management reportedly reduces early rebleeding more efficiently than conservative treatment.^7^^,^^8^ Endoscopic treatment is therefore recommended for definite CDB, which includes various modalities such as epinephrine injection, bipolar coagulation, clipping, and endoscopic band ligation (EBL). Among these modalities, clipping has been widely used because it was considered a simple and minimally invasive treatment.^9^, ^10^, ^11^ Studies reveal that EBL reduces the early and late recurrent bleeding rates of CDB compared with clipping.^12^, ^13^, ^14^, ^15^, ^16^ Perforation is an adverse event of endoscopic treatment that has been reported in addition to subsequent diverticulitis in EBL; however, it was not observed in clipping.^16^, ^17^, ^18^, ^19^ Consequently, EBL is considered more invasive compared with clipping, although no actual pathologic evaluation has yet confirmed this.

We previously performed an animal study comparing the effects of a new EBL device versus an existing EBL device on the colon wall.^20^ At that time, marking clips were performed on the existing EBL device. Therefore, to evaluate the invasiveness of the EBL device and clipping on the colonic mucosa, we evaluated the effects of the existing EBL device and marking clips used in the previous study and performed a new pathologic comparison.

## Methods

Previously, we compared the new EBL device versus the existing EBL device and reported on their feasibility in a pig model, in which clipping was used to mark the procedure site of an existing EBL device.^20^ In the current study, we evaluated the effects of marking clips on the colon wall, which had not been evaluated in our previous study, and compared the effects of existing EBLs and clips on the colon mucosa in a pig model. The experiment was conducted at Fukushima Medical Development Support Centre (Fukushima, Japan). Animal care and experiments were performed according to Guidelines for Proper Conduct of Animal Experiments from the Science Council of Japan. This experiment was approved by the Animal Experiment Committee of Toranomon Hospital.

The procedures were performed on female pigs weighing approximately 40 kg. The pig was fasted on the day of procedures and autopsy. For sedation, a combination of midazolam (8 mg) and medetomidine (1.6 mg) was administered intramuscularly in the neck. Thiamylal sodium (200 mg) was slowly administered intravenously as an anesthetic induction drug. For analgesia, buprenorphine (.4 mg) was administered intramuscularly. Subsequently, the rectum was cleaned with warm water for bowel preparation. The pig was ventilated with tracheal intubation, and anesthesia was maintained by isoflurane (1.5-2.5%) with an inspiratory fraction of oxygen of .4 to 1.0 (oxygen-enriched air).

The procedure was performed by an expert endoscopist (D.K.), a certified specialist by the Japan Gastroenterological Endoscopy Society with extensive experience with >5000 colonoscopies. The EBL procedure was performed between 16 and 30 cm from the anal verge, alternating between a newly developed EBL device and an existing EBL device (Sumitomo Bakelite Co, Ltd, Tokyo, Japan). In the EBL procedure, the colonic mucosa was aspirated into the cap and then ligated with an O-ring. Clipping was performed by using a hemostatic clip (HX-610-135, Olympus Medical Systems, Tokyo, Japan) at about 1 cm, as a marking for each existing EBL device procedure.

### Pathologic analysis

Ten days after the procedure, the pig was sedated, and the procedural sites were observed using a colonoscope. After observation, the animals were anesthetized with 3% to 5% isoflurane and killed with intravenous potassium chloride. Thereafter, the abdomen was accessed and the ileum up till the rectum was resected to further examine each ligation and clipping site of the resected specimen, which were fixed with 10% neutral buffered formalin. Next, hematoxylin-eosin and Masson’s trichrome staining were performed to assess fibrosis. Furthermore, necrosis of the procedure site, disruption of the muscularis mucosae, and fibrosis of the submucosal layer and extending to the serosa were gauged pathologically.

### Statistical analysis

Categorical variables were compared by using the Fisher exact test. Statistical significance was set at *P* < .05. All analyses were performed by using R version 4.1.0 (R Foundation for Statistical Computing, Vienna, Austria).

## Results

**Figure 1:**
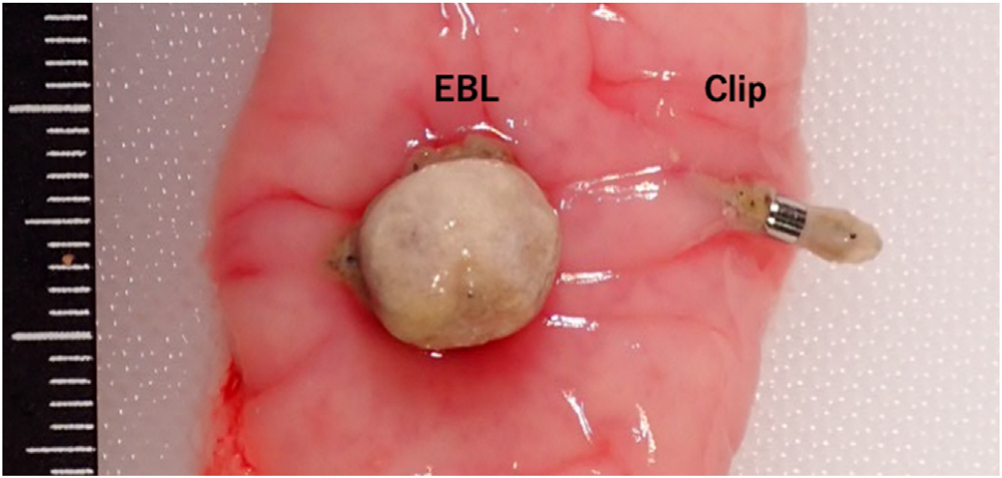
Clipping and endoscopic band ligation (EBL) at autopsy. A ligatured ridge is formed at the EBL site, with no noticeable change at the clipping site.

**Figure 2:**
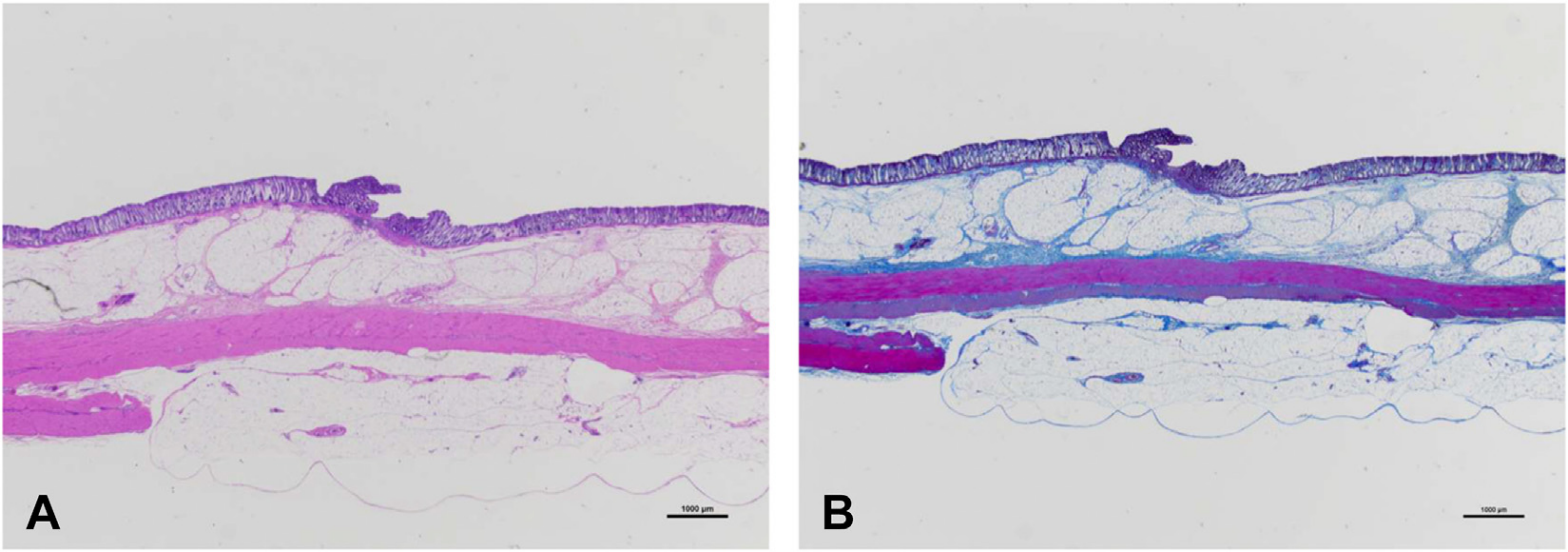
Pathologic evaluation of clipping site. **A,** H&E staining, orig. mag. ×10. **B,** Masson’s trichrome staining, orig. mag. ×10. H&E staining and Masson’s trichrome staining revealed no fibrosis.

**Figure 3:**
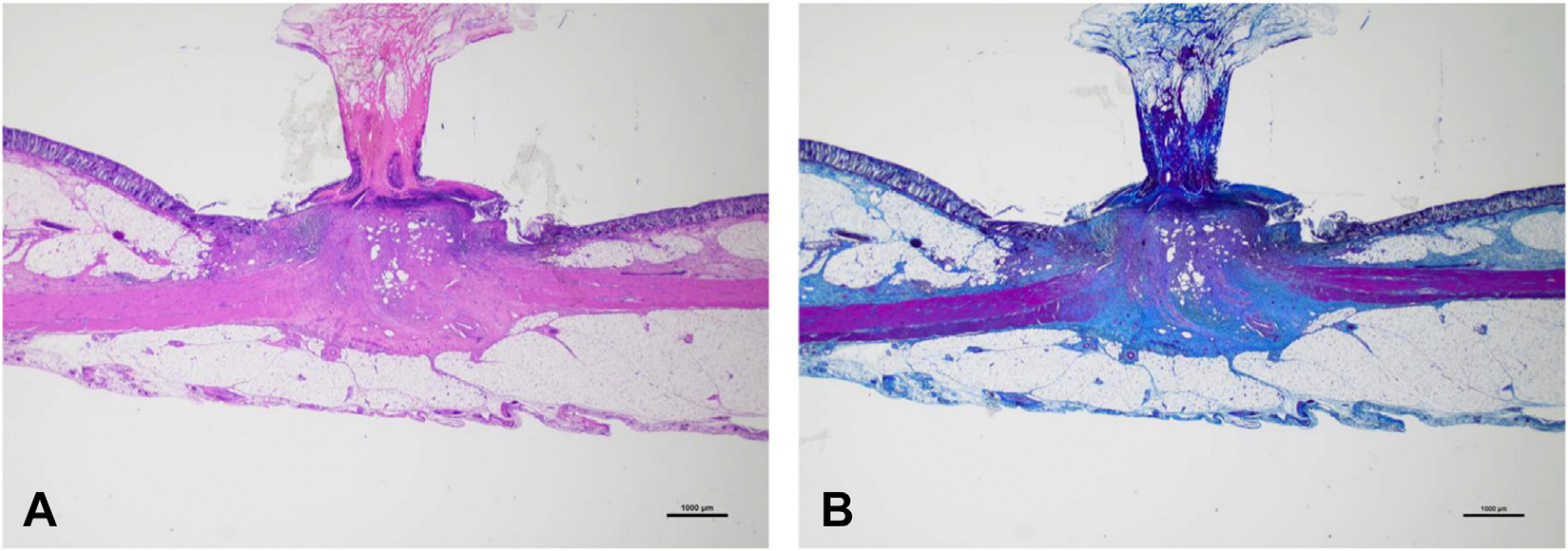
Pathologic evaluation of endoscopic band ligation site. **A,** H&E staining, orig. mag. ×10. **B** Masson’s trichrome staining, orig. mag. ×10. H&E staining shows fibrosis from the submucosal layer to the serosa. Masson’s trichrome staining portrays disruption of the muscularis mucosae and muscularis propria, which were replaced by fibers.

A total of 6 EBL procedures were performed, after which 6 clipping procedures were applied near each EBL site. Both procedures were performed without adverse events. Table 1 presents results of the pathologic evaluation. At autopsy, all O-rings and clips were found intact at the procedural site (Fig. 1). All EBL sites displayed the ligatured ridge, with no noticeable changes observed at the clip sites, and no perforation at either site. Necrosis was significantly less in the clip group but present in all pigs in the EBL group (*P* = .002). In the EBL group, necrosis was observed in the entire ligatured ridge and in >50% of the ligation site. Disruption of the muscularis mucosae was less common in the clip group, but the difference was not significant (Fig. 2). Furthermore, fibrosis of the submucosal layer was significantly less in the clip group but was observed in all cases in the EBL group (*P* = .002). Fibrosis extending to the serosa was less common in the clip group (0 cases [0%] in the clip group and 4 cases [66.7%] in the EBL group) but was not statistically significant (*P* = .061) (Fig. 3). In addition, the muscularis propria at the treatment site was frequently replaced by fibrosis in the EBL group.

**Table 1:** Pathologic results comparing clipping and EBL procedures

Variable	Clipping group (n = 6)	EBL group (n = 6)	*P* value
Necrosis of procedural site	0% (0/6)	100% (6/6)	.002
Disruption of the muscularis mucosae	50% (3/6)	100% (6/6)	.182
Fibrosis of the submucosal layer	0% (0/6)	100% (6/6)	.002
Fibrosis extending to the serosa	0% (0/6)	66.7% (4/6)	.061

*EBL*, Endoscopic band ligation.

## Discussion

To the best of our knowledge, this study is the first to compare the effects of clipping and EBL on colorectal tissue. This study made several findings. First, tissue necrosis is less frequent with clipping, and fibrosis is limited to the mucosal layer and is localized in extent. Second, EBL exhibited more frequent tissue necrosis, with fibrosis extending to the submucosal layer and often to the serosa, along with the muscularis propria being replaced by fibrosis. We speculate that these differences affect the clinical course of CDB with clipping or EBL. Furthermore, clipping was found to be less invasive than EBL. The extent of invasion by clipping is limited and confined to the clipped area, particularly because clipping did not grossly alter the colonic mucosa and only affected the mucosal layer histologically. No perforation has been reported with clipping; however, there have been reports of diverticulitis and sepsis.^16^^,^^21^ Therefore, although clipping is a minimally invasive treatment, monitoring is imperative, and the clip must directly capture the vessel responsible for the bleeding.

There are 2 types of clipping: direct and indirect. Direct clipping captures the blood vessel directly, whereas indirect clipping closes the diverticulum in a zipper fashion.^22^ Both methods report a similar initial hemostatic rate, but the recurrent early and late bleeding rates are lower with direct clipping.^16^ Because most early recurrent bleeding is from the same site,^12^ it is crucial to catch the blood vessel directly. Although infrequent, clipping can cause scarring^12^ and may prevent late recurrent bleeding. Conclusively, for clipping, which has minimal and limited impact on tissue, directly capturing blood vessels is crucial.

In the EBL group, extensive fibrosis was noticed, extending to the serosa in more than one-half of the cases (95% confidence interval, 29-100). However, fibrosis extending to the serosa was more common in the EBL group than in the clip group but without statistical significance. If this study encompassed a larger sample, perhaps this difference would have statistical significance. Moreover, the muscular layer being replaced by fibrosis suggests that it is more invasive than clipping. Previously, EBL has been reported to be unsafe for the right side of the colon because band ligation involves the muscularis propria and serosa of the small intestine and muscularis propria of the right side of the colon.^23^ Perforation and diverticulitis associated with EBL have also been reported.^16^, ^17^, ^18^, ^19^

EBL is relatively safe in pig colon owing to the bound muscle layers replaced by granulation tissues.^24^ The frequency of perforation and diverticulitis is reportedly .18% and .12%, respectively, which is analogous to whole therapeutic colonoscopies in terms of perforation.^16^ Therefore, although EBL is pathologically more invasive, its clinical impact may be less severe. Interestingly, even in a large-scale multicenter cohort study, no perforation was reported with clipping,^16^ indicating that it is less invasive than EBL. The extensive fibrosis due to EBL observed in this study presumedly contributed to the prevention of late recurrent bleeding, corresponding to reports of the late recurrent bleeding rate being lower in EBL than in clipping.^12^^,^^13^^,^^16^ This may be because the strong fibrosis could scar the diverticulum at the source of the bleeding, reportedly observed in 40% of late recurrent bleeding cases after EBL. It is also speculated that because the muscular layer is aspirated and necrotic, it may possibly later be replaced by granulation tissue, thereby eliminating the diverticulum.

The current study offers pathologic reasons supporting the real-world clinical results of clipping and EBL. Because EBL is limited by the inability to aspirate hard or large diverticula along with the need to remove the endoscope once, it necessitates different endoscopic treatment methods depending on the situation.

This study may be applicable to vascular malformations other than colonic diverticular bleeding. Angiodysplasia results in bleeding from dilated veins in the submucosal layer to lamina propria mucosae and is often treated with argon plasma coagulation. If treated with EBL, it may be effective in preventing recurrence by fibrosing the submucosa in addition to primary hemostasis. In fact, some reports have shown that EBL was more effective than with argon plasma coagulation in treating gastric antral vascular ectasia in the stomach.^25^^,^^26^ In Dieulafoy lesions, which are arterial lesions, it may not be possible to accurately identify the bleeding site during active bleeding. In such cases, direct clipping may be difficult, and EBL may be effective for primary hemostasis. In addition, fibrosis of the submucosa layer after EBL may have the potential to prevent recurrence of the lesion. In arteriovenous malformations, the lesion may extend into the serosa, and EBL affecting the serosa may be dangerous. Clipping may be preferable to EBL for primary hemostasis with endoscopic treatment.

The current study has several limitations. First, this was a single-center, small sample size study. Second, the treatment was performed on a normal mucosa in animals; therefore, the results may not necessarily apply to humans. However, because the pig colonic mucosa is similar to the human colonic mucosa, the results of this study may be applicable to humans as well. Third, we did not assess effects on blood vessels. The previous study reported effects on blood vessels in EBL.^26^ Further studies will be expected to evaluate effects on blood vessels. Fourth, this study compared the effect of just a single clip with EBL. Generally, multiple clips are used when clipping diverticular bleeding. However, in this study, we performed evaluations using the marking clips used in our previous study,^27^ and thus we could not evaluate with multiple clips. We therefore may have underestimated the invasiveness of the colon wall. Further studies are expected to evaluate the effect of multiple clips. Lastly, colonic diverticula are pseudodiverticula, which do not include all layers of the colonic wall; the results may not necessarily apply to colonic diverticula. As for clipping, its effect was found to be shallower than the submucosal layer, and we speculate that the results of this study may be applicable to colonic diverticula without a muscularis propria. The results of this study are not necessarily applicable to colonic diverticula because EBL affected the muscularis propria. However, because EBL also ligates the normal colonic mucosa surrounding the diverticula, it is important to evaluate its efficacy in the colonic mucosa without diverticula, and we believe the results of this study may be applicable to colonic diverticula as well.

In conclusion, invasiveness to the colonic mucosa is less and is more localized with clipping, whereas EBL is larger and more extensive. Treatments using diverse invasiveness play a role in the therapeutic management of CDB, corresponding to individual cases.
